# Motif2Mol: Prediction of New Active Compounds Based on Sequence Motifs of Ligand Binding Sites in Proteins Using a Biochemical Language Model

**DOI:** 10.3390/biom13050833

**Published:** 2023-05-13

**Authors:** Atsushi Yoshimori, Jürgen Bajorath

**Affiliations:** 1 Institute for Theoretical Medicine, Inc., 26-1 Muraoka-Higashi 2-Chome, Fujisawa 251-0012, Japan; yoshimori@itmol.com; 2 Department of Life Science Informatics and Data Science, B-IT, LIMES Program Unit Chemical Biology and Medicinal Chemistry, Rheinische Friedrich-Wilhelms-Universität, Friedrich-Hirzebruch-Allee 5/6, 53115 Bonn, Germany

**Keywords:** proteins, ligand binding sites, active compounds, molecular design, sequence motifs, molecular string representation, machine translation, transformer architecture, kinase inhibitors

## Abstract

In drug design, the prediction of new active compounds from protein sequence data has only been attempted in a few studies thus far. This prediction task is principally challenging because global protein sequence similarity has strong evolutional and structural implications, but is often only vaguely related to ligand binding. Deep language models adapted from natural language processing offer new opportunities to attempt such predictions via machine translation by directly relating amino acid sequences and chemical structures to each based on textual molecular representations. Herein, we introduce a biochemical language model with transformer architecture for the prediction of new active compounds from sequence motifs of ligand binding sites. In a proof-of-concept application on inhibitors of more than 200 human kinases, the Motif2Mol model revealed promising learning characteristics and an unprecedented ability to consistently reproduce known inhibitors of different kinases.

## 1. Introduction

In drug design, it is generally attempted to establish relationships between biological targets and chemical matter. This can be accomplished in different ways, directly or indirectly, for example, by using a three-dimensional (3D) structure of a protein as a template for the design of new ligands or by using a set of small molecules with activity against a given target to infer new active compounds by employing principles of molecular similarity. Early attempts to directly associate target and ligand information for predictive modeling date back about 15 years, when combined protein and small molecule representations (descriptors) were designed to generate machine learning models for distinguishing between true and false protein–ligand associations (complexes) [1,2,3]. For such predictions tasks, neural network or support vector machine classification models were derived [1,2,3]. 

With the advent of deep learning in drug discovery, various deep neural network (DNN) architectures were adapted for exploring new design concepts establishing immediate links between target and ligand information. For example, on the basis of protein 3D structures, graph or voxel representations of ligand binding pockets were generated using DNNs and combined with other networks to produce ligand shapes and new compounds consistent with such shapes [4,5,6]. Furthermore, voxel-based representations obtained via convolutional DNNs [5,6] were used to bridge between structure-based design and deep generative modeling by combining convolutional and recurrent neural networks (RNNs) to produce string representations of new compounds [6]. 

Deep generative modeling [7,8] is increasingly used for compound design [9,10,11]. Preferred DNN architectures for generative modeling include RNNs consisting of long short-term memory (LSTM) units [12], which can also be utilized as encoder–decoder frameworks with intermittent latent space [13], and transformer networks with attention mechanisms [14,15]. Such models have originated from natural language processing [16] for addressing machine translation tasks, that is, converting one (input) sequence of characters into another (output) sequence. In chemistry and drug design, these DNN architectures have been adapted as “chemical language models” for various applications, in particular, for chemical reaction modeling and generative compound design [17,18,19,20]. They depend on the use of textual representations of small molecules, for which “simplified molecular input line entry system” (SMILES) strings [21] continue to be the most widely employed format.

In a few studies, RNNs or transformers have also been applied to associate protein and ligand representations. Specifically, three studies have attempted to generate new small molecule ligands from target protein sequences via language models [22,23,24]. Hence, in these cases, the machine translation task required the derivation of models to construct SMILES representations encoding new compounds from amino acid sequences of targets. In the first study [22], an approach from image processing for generating image captions was adapted [25]. Therefore, a DNN was employed to generate protein sequence vector embeddings [26] that served as input for an RNN comprising multiple LSTM units to generate SMILES strings of new compounds via reinforcement learning [22]. In addition, two methodologically distinct studies trained transformer networks to directly associate protein sequences with SMILES of known compounds and generate new molecules [23,24]. Therefore, a transformer architecture with an attention mechanism was adapted [14,23]. Furthermore, a transformer variant was developed based on the Lmser network [27] to combine embeddings of protein sequences and molecule strings and generate new compounds conditioned on given protein sequences via Monte Carlo tree search over intermittent strings [24]. Both transformer-based approaches used conventional protein–ligand docking scores to assess or the guide compound design [23,24], which increased the intrinsic uncertainty of the design approach (that is, one hypothetical model was employed to guide another). 

In this work, we report the development and application of a simpler transformer model for the design of new active compounds from sequence data. Since the transformer relates amino acid sequences to molecular structure, it is termed a “biochemical language model”. Different from the earlier studies [22,23,24] that learned from complete protein sequences, we use sequence motifs defining ligand binding regions as input to avoid redundancy or noise of sequence information that might not be relevant for ligand binding. In addition, we apply a compound evaluation scheme that does not rely on hypothetical scoring, but directly assess the ability of a model to produce active compounds. As a proof-of-concept application, we design candidate compounds for ATP site-directed protein kinase inhibitors (PKIs) [28,29,30], representing a major class of drug candidates and approved drugs [30]. 

## 2. Materials and Methods 

### 2.1. Methodological Concept

The Motif2Mol approach was designed to generate chemical structures from different amino acid sequence segments (motifs). The underlying idea was to derive a language model that associates sequence signatures of target proteins with specifically active compounds and use the model to predict new compounds for targets (sequence motifs) of interest. For this purpose, a transformer model was implemented.

### 2.2. Model Architecture

**Figure 1:**
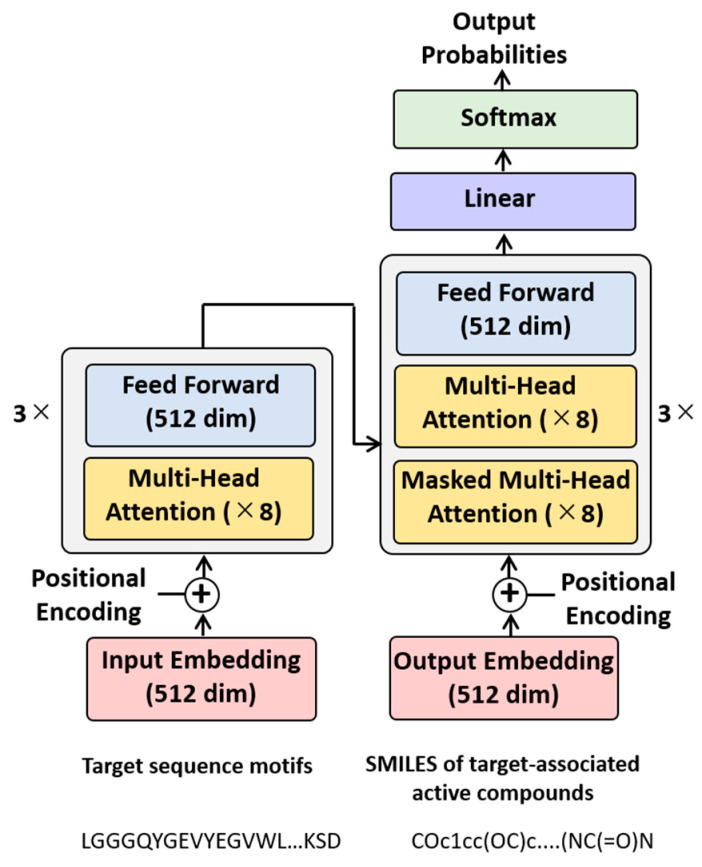
Motif2Mol transformer. Shown is a schematic representation of the architecture of the Motif2Mol transformer specifying multiple units and sub-layers as well as the dimensions (dim) of the input and output embeddings and feed-forward sub-layer.

The choice of the transformer architecture instead of RNNs for this machine translation task involving different types of molecular representations was motivated by the availability of the transformer-specific attention mechanism that makes it possible to operate on hidden states of different parts of input sequences in parallel [14]. Transformers consist of multiple encoder and decoder modules, including attention sub-layers. Each module combines a multi-head self-attention sub-layer and a fully connected feed-forward sub-layer. In a multi-head self-attention sub-layer, several attention functions act on different parts of sequences simultaneously. Figure 1 schematically represents the architecture of the Motif2Mol transformer model. 

The encoder (left) consists of three modules with eight multi-head attention sub-layers each and a feed-forward sub-layer (512 dimensions), which generates a 512-dimensional vector embedding of input sequence motifs through positional encoding (which ensures that the sequential information is retained). The embedding represents the hidden states. The decoder (right) also comprises three modules with multiple attention sub-layers and a feed-forward sub-layer. Here, however, each module contains two types of attention sub-layers (with eight sub-layers of each type). The multi-head attention sub-layers corresponding to those in the encoder operate on encoder-generated hidden states as well as the output of the first decoder module. Thereby, the multi-head attention sub-layers can learn relationships between sequence encodings on the encoder side and structure encodings on the decoder side and pay attention (that is, assign importance) to particular sequence segments based on structural features (and vice versa). This architecture facilitates an effective use of the self-attention mechanism. By contrast, the masked attention sub-layers representing the second type only operate on the output of the preceding attention sub-layer of the decoder modules. The masked attention sub-layers identify (and mask) transmitted information that should not be utilized to ensure that translated encodings are created in the correct sequential order. Hence, these layers are designed to prevent translation errors. SMILES tokens are sampled according to the probability distribution learned by the model. Output probabilities are derived in the softmax layer and the decoder generates a 512-dimensional embedding of the output sequence via positional encoding (corresponding ot the encoder).

### 2.3. Proof-of-Concept Application

**Figure 2:**
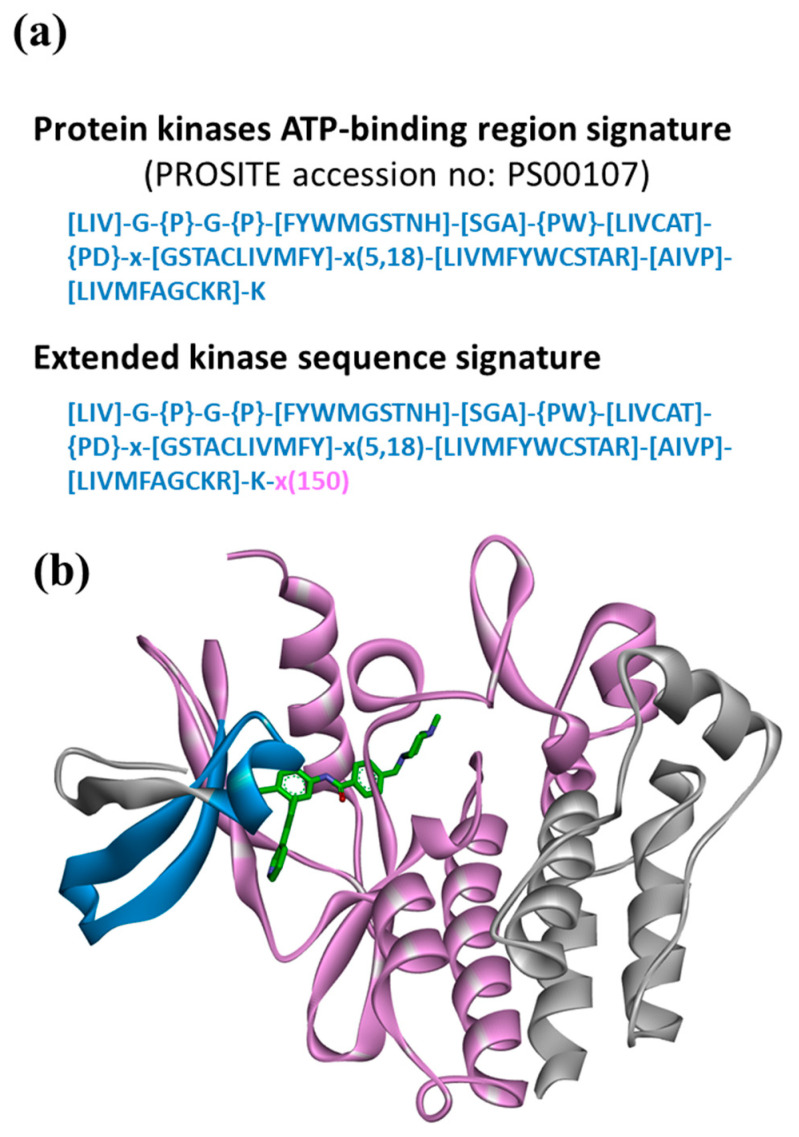
Protein kinase sequence motifs. In (**a**), the PROSITE character string encoding the protein kinase ATP-binding region signature and the extended kinase sequence signature are shown. The character string represents 21–34 residues forming the ATP-binding region in kinases. Alternative amino acids permitted at a given position are indicated by square brackets ‘[]’. For example, [LIV] represents Leu, Ile, or Val at a given position. Amino acids excluded at a position are indicated by curly brackets ‘{ }’. For example, {PW} prohibits Pro and Trp at this position (but permits any other amino acid). ‘x’ accepts any amino acid at a given position and values in parentheses ‘( )’ define sequence ranges. For example, ‘x(5,18)’ defines a sequence segment comprising 5 to 18 residues where any amino acids are permitted at each position (for further details, see PROSITE accession number PS00107). In the extended sequence signature, ‘x(150)’ denotes a sequence segment comprising 150 residue positions (where any amino acids are permitted at each position) added to the PROSITE signature. In (**b**), the PROSITE-encoded ATP-binding region (blue) and the 150-residue extension (magenta) are mapped on a ribbon representation of the catalytic domain of Abl kinase in complex with an ATP site-directed inhibitor (Protein Data Bank ID 2HYY).

As a proof-of-concept application for the Motif2Mol approach, we selected the design of candidate compounds for ATP site-directed PKIs based on kinase sequence motifs: a topical drug discovery task. Figure 2a depicts a character string from PROSITE [31] encoding the sequence signatures of the kinase ATP-binding region. The narrowly defined ATP-binding region comprises 21–34 amino acid residues and was further extended with the following segment of 150 residues, forming an extended kinase sequence signature. The resulting sequence motifs contained kinase-specific sequence information beyond the narrowly defined ATP-binding region while excluding essentially invariant regions of the catalytic kinase domain, as illustrated in Figure 2b. The extended kinase sequence signature was expected to include most residues relevant for the binding of ATP site-directed PKIs. 

**Figure 3:**
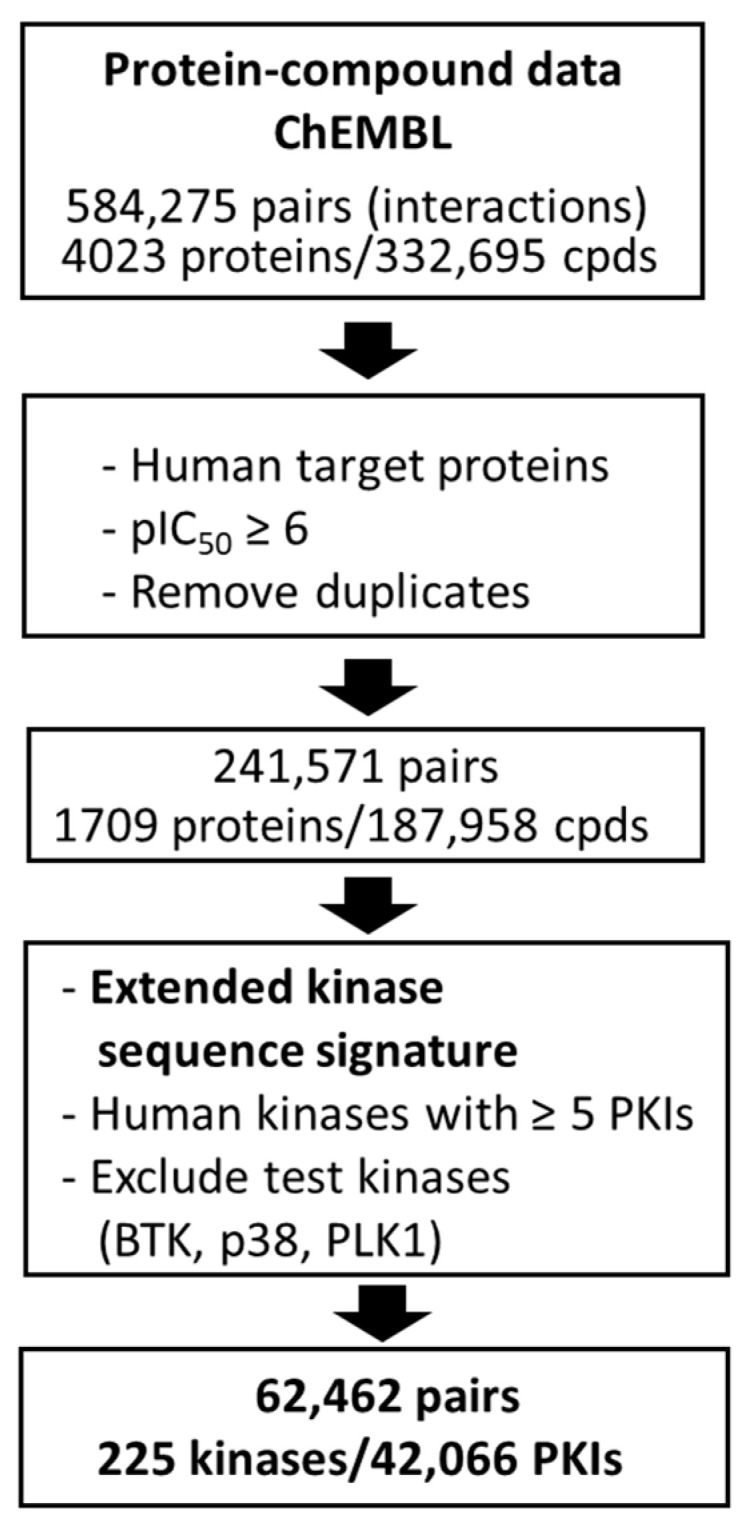
Training and test data. A workflow diagram summarizes the curation of training and test data from ChEMBL (cpds stands for ‘compounds’ and PKIs for ‘protein kinase inhibitors’).

So-defined sequence motifs of human kinases and inhibitors of these kinases were extracted from ChEMBL [32] (version 29). The data curation process is summarized in Figure 3. It ultimately yielded 225 kinases, with a total of 42,066 inhibitors at the highest target confidence level (target confidence score: 9) and with pIC_50_ potency values of 6 or larger. Pairs of 225 sequence motifs and corresponding PKIs were used as input and output for Motif2Mol model derivation and validation, respectively. In addition, three qualifying kinases (BTK, p38, and PLK1) and their inhibitors were exclusively used as test kinases for model evaluation. 

### 2.4. Model Derivation

The Motif2Mol transformer architecture depicted in Figure 1 was implemented using Pytorch [33] based on code available in the “Language Translation with NN.Transformer and Torchtext” section of the Pytorch tutorial [34]. Sequence motif and SMILES tokens were embedded in 512 dimensions, respectively. For the 225 kinases, all possible pairs of a kinase sequence motif and corresponding PKIs were enumerated, pooled, and randomly divided into training (80%) and validation (20%) data. Model training was carried out over 100 epochs using a batch size of 32. The Motif2Mol model was trained on a NVIDIA GeForce RTX 2080 Ti GPU for approx. three hours.

### 2.5. Generation of New Candidate Compounds

For the generation of new PKI candidate compounds, SMILES tokens were sampled according to the learned probability distribution of the Motif2Mol transformer. To evaluate the sampling characteristics and output of the Motif2Mol model based on training and validation data, sampling runs were performed at temperature T = 1.0 until 100 unique candidate compounds were generated for each kinase. Furthermore, to evaluate Motif2Mol on test kinases, 1000 sampling runs were carried out at T = 1.0 modifying the probability distribution for the sampling of the tokens [35]. The calculation time for 1000 sampling runs for structure generation was ~4.7 min on an Intel Core i9-9900K CPU. Compound structures generated using the Motif2Mol transformer were assessed using the following metrics.

Validity was used as a metric to quantify the proportion of chemcially correct (valid) structures among all generated SMILES strings. It is defined as the ratio N_valid_/N_all_, where N_valid_ is the number of valid structures, as assessed using RDKit [36], and N_all_ the total number of generated SMILES strings. 

Maximum 1-nearest neighbor (1-NN) similarity (Equation (1)) and average 1-NN similarity (Equation (2)) were calculated to compare the newly generated structures (set of structures A) and existing inhibitors (set of structures B) of a target kinase.

(1) $$1NN\;Sim^{max}\left(A,B\right)=\underset{a\in A}{\max}(\underset{b\in B}{\max}\left(T_{c}\left(a,b\right)\right))$$

(2) $$1NN\;Sim^{ave}\left(A,B\right)=\frac{1}{\left|A\right|}\;\underset{a\in A}{\sum }\underset{b\in B}{\max}\left(T_{c}\left(a,b\right)\right)$$

where *Tc* is the Tanimoto coefficient [37] and |*A*| represents the number of structures in set *A*. The *Tc* was calculated using 2048-bit Morgan fingerprints of radius 3 [38] of structures *a* and *b*.

### 2.6. Sequence Comparison

Sequence identity between two kinases was calculated via pairwise sequence alignment using the *pairwise2* function implemented in BioPython [39] using *BLOSUM62* [40] as the scoring matrix.

## 3. Results and Discussion

### 3.1. Motif2Mol Model Evaluation and Performance 

**Figure 4:**
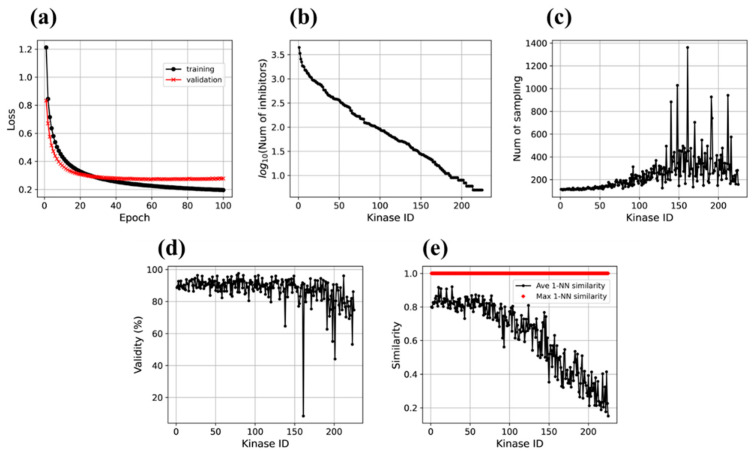
Evaluation of the Motif2Mol model based on training and validation data. (**a**) shows learning curves with training and validation losses over 100 epochs, (**b**) the number of known inhibitors of each of the 225 kinase targets (with kinase identifiers (IDs) arranged in the order of decreasing numbers of available PKIs), (**c**) the number of sampling runs required to generate 100 unique candidate compounds for each kinase, (**d**) the proportion of valid SMILES strings produced over all sampling runs, and (**e**) the average (ave) and maximal (max) 1-NN similarity of the 100 newly generated compounds compared to known inhibitors of each kinase.

To establish proof-of-concept of the approach, a large-scale investigation on sequence motifs and PKI data of 228 human kinases was carried out. The Motif2Mol transformer was trained on 49,969 sequence motif/PKI pairs (80%). The trained model was then evaluated using 12,493 sequence motif/PKI pairs (20% validation data). Figure 4a compares the training and validation loss over 100 epochs, which accounts for the sum of errors over all training and validation instances, respectively, after each iteration. Both training and validation loss sharply decreased over the first iterations and became essentially constant (validation loss) or nearly constant (training loss) at a low loss level after ~40 epochs. During training, the loss further decreased slightly over the remaining epochs. The evolution of training and validation loss over 100 epochs indicated that the Motif2Mol quickly reached a high level of prediction accuracy. Validation loss remained constant and comparable to training loss, hence providing no indications for potential model overfitting. 

In Figure 4b, the 225 kinases are ordered according to decreasing numbers of available PKIs, corresponding to decreasing volumes of training data, confirming that the number of known inhibitors significantly varied among the large number kinases, as one would expect. Accordingly, model derivation should become increasingly difficult as amounts of training data decrease. Figure 4c reports the number of sampling runs required to generate 100 unique valid compounds for each kinase arranged in the same order. For the first 50 kinases with largest numbers of available training instances, only ~100 runs were required and for most of the first 100 kinases, 200 or fewer runs. Then, the number of runs gradually increased to ~400. For the ~100 kinases with the smallest amounts of available training data, only a few outliers with much larger numbers of runs were observed, but also a number of kinases for which only less than 200 sampling runs were required. Taken together, these observations not only revealed an expected (moderate) loss of structure generation frequency for decreasing amounts of available training data, but also an overall stable structure generation capacity for the Motif2Mol model. 

### 3.2. Validity of Generated Molecular Representations

In addition to studying learning curves and structure generation frequency, analyzing the validity of generated molecular representations (see Section 2.5) represented another relevant measure of model performance. Figure 4d reports the proportion of valid SMILES representations among all SMILES strings generated using the Motif2Mol model over all sampling runs for all ordered kinases. With the exception of a few outliers among kinases with smallest numbers of training compounds, the quality of molecular representations generated using the Motif2Mol model was generally high (the calculations essentially failed for only a single kinase). For the first 100 kinases, consistently more than 80%, and often close to 100%, of the generated SMILES strings were valid (with only one exception). For the next 100 kinases, the proportion of valid SMILES only slightly decreased, and even for the majority of kinases with the smallest numbers of training compounds, the proportion of valid SMILES strings remained at or close to the 80% level. 

### 3.3. Similarity Analysis

We then systematically determined the similarity of newly generated Motif2Mol compounds to PKIs. The results reported in Figure 4e reveal another clear trend for average nearest neighbor similarity. For ~70 kinases with largest amounts of available training data, the average 1-NN similarity between newly generated and known compounds was consistently high, at or above the 80% level, and then monotonically decreased with decreasing amounts of training data to less than 20% for kinases with the fewest training instances. The correlation between decreasing average similarity and decreasing amounts of training data indicated that the ability of the transformer to produce structures with varying levels of similarity to known compounds could be controlled by adjusting the number of training instances; an interesting feature for model derivation and tuning. The ability of the Motif2mol model to generate increasingly similar or diverse structures relative to known compounds can be easily monitored based on average nearest neighbor similarity calculations, as shown in Figure 4e. The average 1-NN similarity of 100 newly generated structures to known PKIs tended to decrease with decreasing numbers of PKIs (for each of the first 50 kinases, more than 300 known PKIs were available and for each of the last 50 kinases, less than 15 known PKIs). Statistically, the average 1-NN similarity between a constantly sized set of candidate compounds and increasing numbers of known PKIs is likely to increase. This is the case because for each new structure, increasing numbers of reference compounds are available for pairwise comparison that do not represent a structurally diverse sample but tend to be similar (since they are active against the same target). This statistical tendency is observed in Figure 4e. However, potential contributions of training bias due to increasing numbers of related PKIs that might limit the diversity of newly generated structures can principally not be excluded.

Furthermore, maximal nearest neighbor similarity calculations revealed that new structures with 100% fingerprint similarity to known inhibitors were generated for all kinases, regardless of the amounts of available training data (giving rise to the apparent horizontal red bar at the top of Figure 4e that is formed by adjacent diamond symbols). We note that 100% fingerprint similarity defines pairs of identical or nearly identical compounds. Thus, the Motif2Mol transformer consistently reproduced known PKIs across the 225 kinases for both training and validation data. 

### 3.4. Predictions for Test Kinases

The ability of a generative model to reproduce known active compounds such as PKIs represents the best possible criterion for model performance prior to prospective applications. Therefore, in addition to training and validation sets of sequence motif/PKI pairs, the Motif2Mol model was also applied to predict candidate PKIs of three test kinases in independent trials that were not encountered during the training or validation phase. The test kinases included the popular drug targets BTK, p38, and PLK1 that were selected based on varying global sequence identity to training set kinases. 

**Figure 5:**
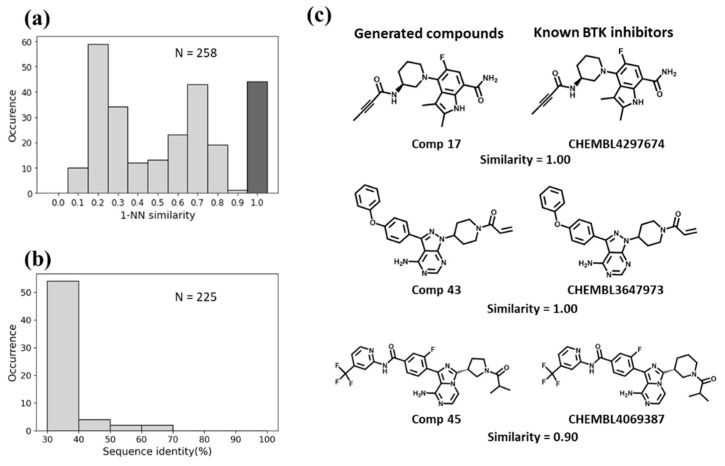
Evaluation of the Motif2Mol model based on BTK test data. (**a**) shows the distribution of 1-NN similarities of 258 unique candidate compounds generated in 1000 sampling runs compared to 1382 known BTK inhibitors, (**b**) the distribution of sequence identities of BTK compared to 225 training kinases, and (**c**) exemplary newly generated compounds and the most similar known BTK inhibitors (with ChEMBL IDs). For each pair of newly generated compounds and PKIs, the fingerprint Tanimoto similarity value is reported. In addition, the “Comp x” label gives the position of the compound pair in the ranking of Motif2Mol candidate PKIs according to its maximal nearest neighbor similarity to known inhibitors.

**Figure 6:**
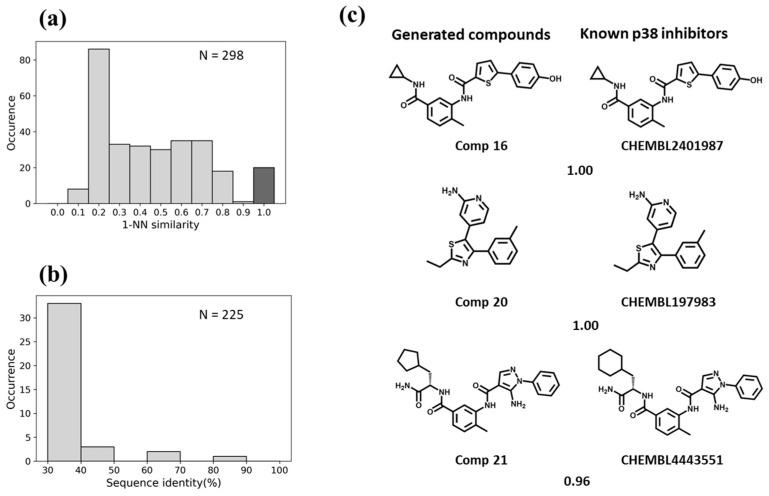
Evaluation of the Motif2Mol model based on p38 test data. (**a**) shows the distribution of 1-NN similarities of 298 unique candidate compounds generated in 1000 sampling runs compared to 1808 known p38 inhibitors, (**b**) the distribution of sequence identities of p38 compared to 225 training kinases, and (**c**) exemplary newly generated compounds and the most similar known p38 inhibitors, represented according to Figure 5c.

**Figure 7:**
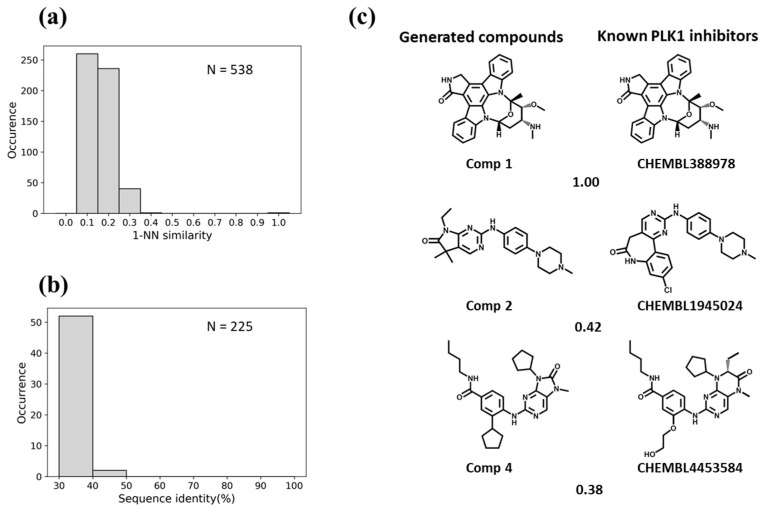
Evaluation of the Motif2Mol model based on PLK1 test data. (**a**) shows the distribution of 1-NN similarities of 538 unique candidate compounds generated in 1000 sampling runs compared to 275 known PLK1 inhibitors, (**b**) the distribution of sequence identities of PLK1 compared to 225 training kinases, and (**c**) exemplary newly generated compounds and the most similar known PLK1 inhibitors, represented according to Figure 5c.

For BTK, the Motif2Mol model generated 258 unique candidate compounds covering a wide range of 1-NN similarities to known BTK inhibitors, ranging from distinct structures (10–20% similarity) to identical structures (Figure 5a). BTK displayed a sequence identity of 50–70% to four training set kinases (Figure 5b). The Motif2Mol model exactly reproduced 44 known BTK inhibitors (Figure 5a). Representative examples of new candidate compounds and known BTK inhibitors are shown in Figure 5c. For p38, the model sampled 298 candidate compounds that also covered a wide range of 1-NN similarities to known PKIs (Figure 6a). Kinase p38 displayed 60% to more than 80% sequence identity to three training set kinases (Figure 6b). The model reproduced 20 known p38 inhibitors. Representative examples are shown in Figure 6c. For PLK1, a total of 538 candidate compounds were obtained that were mostly dissimilar to known inhibitors (Figure 7a). For PLK1, no training set kinase with more than 50% sequence identity was available (Figure 7b). More than 500 candidate compounds were successfully sampled in this case, and one of 275 known PLK1 inhibitors was exactly reproduced, as shown in Figure 7c. Hence, for all three test kinases, the Motif2Mol model successfully reproduced known PKIs, indicating its capacity to predict active compounds based on sequence motifs. 

Taken together, the findings discussed above show that the Motif2Mol model consistently reproduced known PKIs for all 225 training/validation kinases and three test kinases not encountered during the training and validation phase. Thus, the results provide substantial support for the ability of the Motif2Mol model to generate new specifically active compounds. 

## 4. Conclusions

In this work, we have addressed the design of new active compounds from protein sequence data by considering this design effort as a machine translation task. Accordingly, machine learning was used to transform amino acid sequences into different sequences of tokens representing chemical structures. Therefore, a transformer network was derived to associate sequence motifs of binding site regions in target proteins with textual ligand representations and predict new candidate compounds from sequence motifs. For establishing proof-of-concept, the pilot version of the Motif2Mol transformer was implemented exclusively using public domain programs and available code [33,34,36], as specified above, making it fully reproducible based on the methodological information provided herein. In a large-scale proof-of-concept application, this biochemical language model was applied to inhibitors of a total of 228 human protein kinases. The Motif2Mol model exhibited favorable learning characteristics with closely corresponding training and validation loss, reflecting a high level of accuracy and consistent generation of valid compound representations for varying amounts of available training data. We reasoned that reproduction of known inhibitors of different kinases represented a rigorous criterion for model validation, taken into consideration that it is typically difficult to exactly reproduce known active compounds using generative models. An underlying reason for this is the vastness of chemical space surrounding islands of compounds with activity against given protein targets or families. Importantly, however, the Motif2Mol model consistently reproduced varying numbers of known inhibitors for all investigated kinases, including test kinases not encountered during training and initial validation. Taken together, the findings reported herein suggest that the prediction of novel active compounds from sequence motifs of pharmaceutical targets via language models complements and further extends structure and ligand similarity-based approaches currently used in drug design. Having established proof-of-concept for the approach in the current investigation, subsequent Motif2Mol applications will focus on compound design for other pharmaceutical target classes. Notably, this might require the design of new or further refined sequence motifs for active sites or ligand binding sites in different targets. Defining such sequence motifs generally depends on prior knowledge of active or ligand binding sites as well as compound binding or inhibition characteristics and can thus be challenging. On the basis of these studies and depending on their results, further methodological refinements of the Motif2Mol approach can be considered.

## Data Availability

Compounds and activity data were obtained from the publicly available ChEMBL database (https://www.ebi.ac.uk/chembl/, accessed on March 5, 2023). For inquiries concerning the current version of Motif2Mol, please contact the authors.
